# Metaplastic Breast Carcinoma Presenting as a Mixed Solid and Cystic Lesion

**DOI:** 10.7759/cureus.22029

**Published:** 2022-02-08

**Authors:** Hassan Arekemase, Osmani Mohammed, Usma Zafar, Kohli Manpreet, Amin Maghari

**Affiliations:** 1 Anatomical and Clinical Pathology, Saint Barnabas Medical Center, Livingston, USA; 2 Radiology, Monmouth Medical Center, Long Branch, USA; 3 Pathology and Laboratory Medicine, Saint Barnabas Medical Center, Livingston, USA; 4 Surgery, Monmouth Medical Center, Long Branch, USA; 5 Pathology, Community Medical Center, Toms River, USA

**Keywords:** breast cancer, general surgery and breast cancer, breast cancer biology, breast cancer pathology, breast and gynaecological pathology, metaplastic breast cancer

## Abstract

The objective of this paper is to report a rare presentation of metaplastic breast carcinoma (MBC) as a mixed solid/cystic mass and emphasize the possibility of having a different biopsy report and final diagnosis.

MBC is a rare type of breast cancer consisting of mesenchymal or mixed mesenchymal and epithelial components. It is believed to result from the conversion of glandular cells into non-glandular cells through various forms of mutations. It is common in females and more frequent in older women. MBC is a triple-negative breast cancer (progestin receptor, estrogen receptor, and human epidermal growth factor receptor 2) with a very poor prognosis when compared with other types of breast cancers.

This is a case of an 84-year-old woman presenting with rapidly growing mixed solid and cystic breast mass with a final diagnosis of MBC. The initial biopsy report was high-grade ductal carcinoma in situ. This is an unusual presentation of MBC.

## Introduction

Breast cancer is the second most common cause of cancer-related deaths affecting the female population in the United States [1,2]. Metaplastic breast carcinoma (MBC) is a very rare variant of breast cancer, accounting for about 1% of all breast cancers [3,4]. MBC is defined as a group of variable breast diseases with mesenchymal or mixed mesenchymal and epithelial components [5,6]. It is a very rare type of breast cancer with a poor prognosis [7,8].

According to the World Health Organization (WHO) 2019 classification of MBC, a few variants were identified: squamous cell carcinoma, low-grade adenosquamous carcinoma, spindle cell carcinoma, fibromatosis-like carcinoma, MBC with heterologous mesenchymal differentiation, and mixed MBC [3,9].

The presentation is variable but mostly presents as a well-circumscribed or irregular solid mass. Occasionally, calcifications are identified in MBC with heterologous mesenchymal differentiation variants [4].

## Case presentation

An elderly woman presented with fatigue, weight loss, and a 10-cm palpable right breast mass without any significant lymphadenopathy. The mass was painless, firm, and slightly mobile. No nipple discharge and no skin discoloration were noted. She had a past medical history of hypertension, end-stage renal disease on dialysis, and hip replacement. She denied fever, headache, blurring of vision, palpitation, chest pain, cough, breathing difficulties, change in bowel habits, back pain, and urinary symptoms. She had no family history of cancer, was not an alcoholic, quit smoking 25 years ago, and had no illicit drug use. She is married in a monogamous setting with three children and good family support.

**Figure 1:**
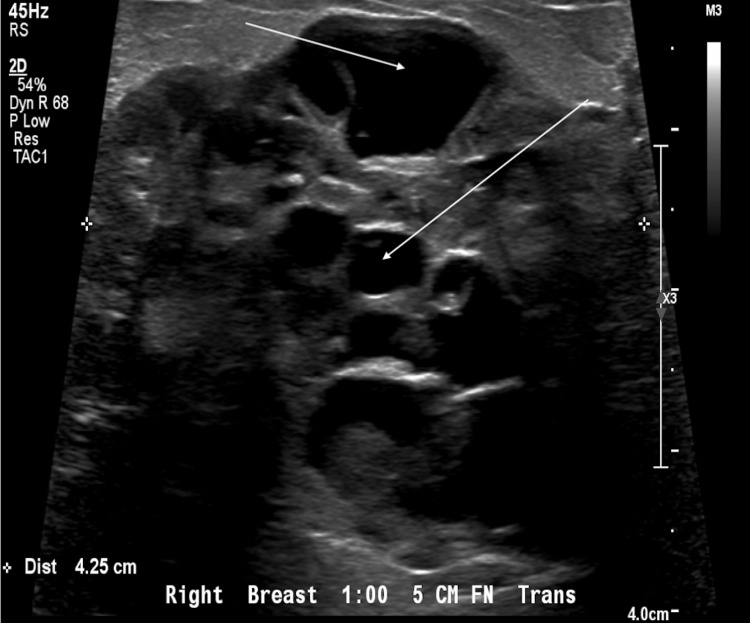
Ultrasound - transverse view. Arrows pointing to the cystic spaces.

Mammography reveals large lobulated low-density soft tissue mass in the right breast with relatively well-defined margins, the largest in the subareolar region. Breast ultrasound showed heterogeneous lobulated mixed solid and cystic mass in the right breast at the 1 o’clock position, 5 cm from the nipple (Figure 1).

**Figure 2:**
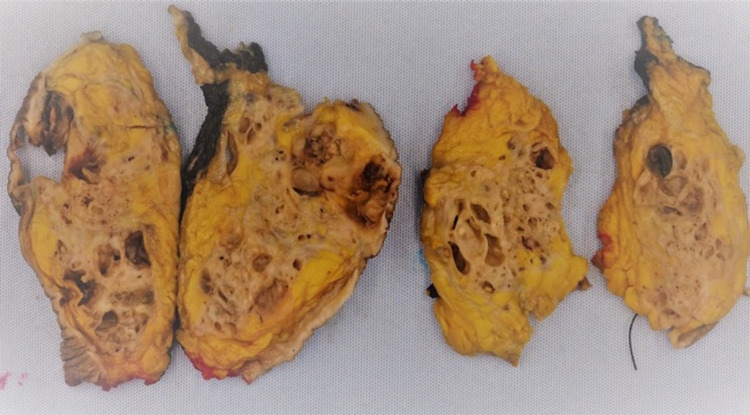
Formalin-fixed breast resection. Gross appearance of brown-red tan solid and cystic lesion in a background of yellow tan fibrofatty breast tissue.

**Figure 3:**
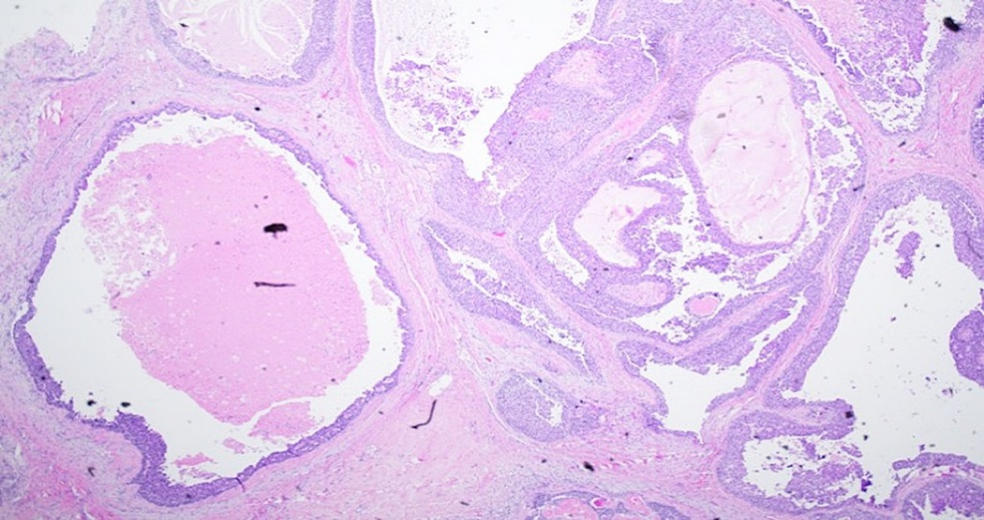
Low power image of solid and cystic areas. Hematoxylin and eosin stain revealing cystic spaces with in situ, comedonecrosis, and invasive carcinoma.

**Figure 4:**
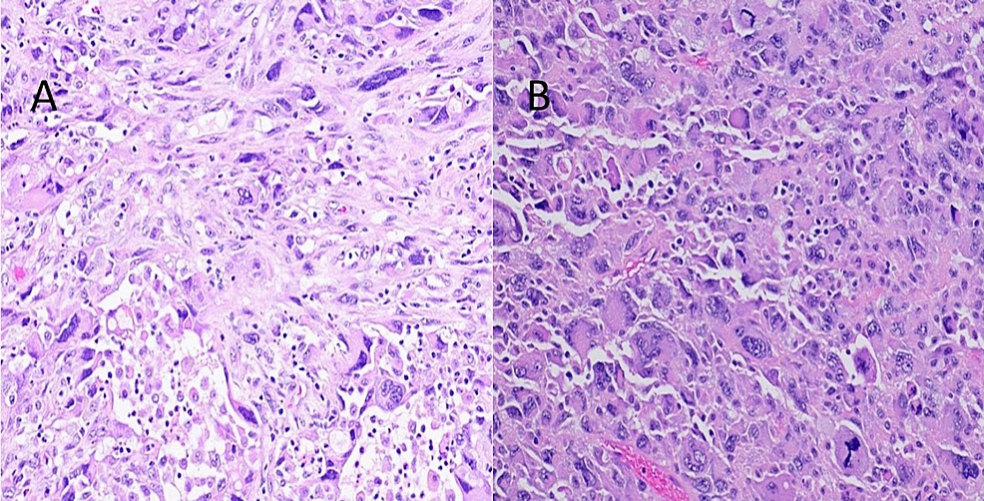
(A) Hematoxylin and eosin stain showing poorly differentiated tumor cells with pleomorphism, mitosis, and atypical spindle cell morphology. (B) Hematoxylin and eosin stain showing giant cells, pleomorphism, and mitosis.

**Figure 5:**
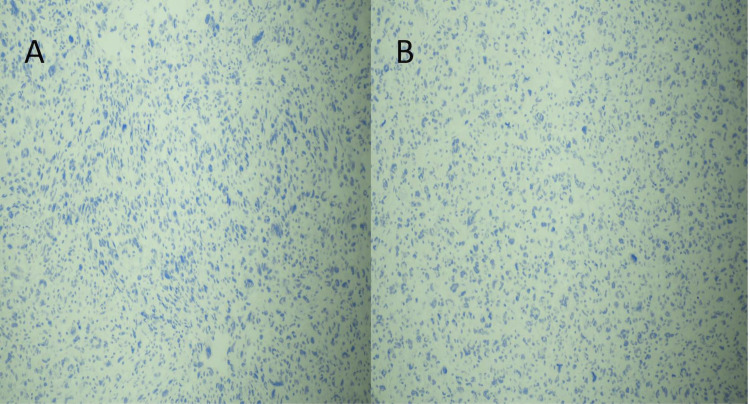
Immunohistochemistry: (A) estrogen receptor and (B) progesterone receptor. Immunohistochemical stain showing a negative estrogen and progesterone receptor.

The biopsy was reported as multiple fragments of ductal carcinoma in situ, high grade with comedonecrosis. The resection specimen revealed a 10 x 10 cm multilocular cystic and solid mass with necrosis and exudates (Figure 2). The light microscopy showed invasive carcinoma, high-grade carcinoma in situ, pleomorphism, mitosis, atypical spindle cell proliferation, and giant cells (Figures 3, 4). The epithelial and spindle cell differentiation was positive for cytokeratin and OSCAR. Hormone receptor studies were all negative (Figure 5).

## Discussion

Metaplastic carcinoma of the breast was first described by Huvos in 1973 [10]. National Institute of Health classifies metastatic carcinoma of the breast as a rare disease [11]. The incidence is 0.2-5 % of all breast cancer cases and the average age of presentation is 55 years [12]. Clinically, it presents as a well-circumscribed palpable mass and is a well-delineated mass radiologically. The presentation is the same as invasive ductal carcinoma and not otherwise specified [12]. However, in our case, the lesion is a mixed cystic and solid. This is unusual and an important pattern to recognize.

The components of a metaplastic carcinoma can be epithelial, mesenchymal, or combined [13]. According to Huina Zhang, the epithelial-only carcinoma can be squamous cell carcinoma or adenosquamous carcinoma while the mesenchymal-only carcinoma can be fibromatosis-like metaplastic carcinoma and spindle cell carcinoma. Other elements present singly or in combination can be chondroid, osseous, rhabdomyosarcomatous, angiosarcomatous, liposarcomatous, and neuroglial differentiation [14].

The spindle cell component may vary from spindle cell sarcoma to malignant fibrous histiocytoma-like sarcoma and can have syncytiotrophoblast-like giant cells [15]. Depending on the components present, the differential diagnosis can be quite variable including phyllodes tumor, primary breast sarcoma, myoepithelial carcinoma, metastatic sarcoma, adenomyoepithelioma, and pleomorphic adenoma. Wong et al. also suggested lobular carcinoma, pleomorphic carcinoma, and rare sarcomas such as angiosarcoma as differential [16].

The histological components in metaplastic carcinoma can be heterologous and immunohistochemistry is an essential tool in challenging cases. MBC is positive for basal cytokeratin including cytokeratin 5/6 (CK5/6) and 34BetaE12 [17]. According to Koker et al., p63 has a high sensitivity and specificity (86.7% and 99.4%, respectively), and it may stain both the epithelial and spindle cell component. They suggested p63 may aid in the diagnosis of spindle cell tumors of the breast [18].

Richard et al. identified positivity of different markers helpful in the diagnosis of MBC: laminin 5 (96%), p63 (57-86%), CK5/6 (50-86%), CD10 (85%), smooth muscle actin (SMA) (60%), and S-100 (45%) [19]. The most important point to remember is that the presence of SMA and S-100 positivity does not rule out carcinoma. This is a fallout from the basic principle of breast pathology in which the presence of SMA, a myoepithelial marker, suggests a benign process. Han et al. have suggested the combination of OSCAR, cytokeratin 14 (CK14), and p63 is the most efficient panel (sensitivity 97.9%) for diagnosing MBC [2].

MBC has a poorer prognosis as compared to other triple-negative breast carcinomas (TNBC). In a study by Song et al., the five-year overall survival rate is 54.5% (MPC) vs. 73.3% (TNBC). The study also concludes that the prognosis differs with the cellular component present in the MPC. Adenocarcinoma with spindle cell differentiation had the worst five-year overall survival rate at 40% [20].

## Conclusions

For pathologists, extensive sampling of triple-negative breast lesions is important as there might be hidden metaplastic components which will impact the patient's survival rate and management. For radiologists, MBC can also present as a mixed solid and cystic lesion. A high index of suspicion and adequate biopsy sampling is important. For surgeons, even though the biopsy report read invasive or in situ carcinoma, resection specimen might reveal a different diagnosis due to extensive specimen sampling.
